# Animal Models of Anxiety and Depression: Incorporating the Underlying Mechanisms of Sex Differences in Macroglia Biology

**DOI:** 10.3389/fnbeh.2021.780190

**Published:** 2021-12-09

**Authors:** Amy J. Wegener, Gretchen N. Neigh

**Affiliations:** Department of Anatomy & Neurobiology, Virginia Commonwealth University, Richmond, VA, United States

**Keywords:** glia, astrocytes, oligodendrocytes, stress, anxiety, depression, sex differences

## Abstract

Animal models have been utilized to explore the mechanisms by which mood disorders develop. Ethologically based stress paradigms are used to induce behavioral responses consistent with those observed in humans suffering from anxiety and depression. While mood disorders are more often diagnosed in women, animal studies are more likely to be carried out in male rodents. However, understanding the mechanisms behind anxiety- and depressive-like behaviors in both sexes is necessary to increase the predictive and construct validity of the models and identify therapeutic targets. To understand sex differences following stress, we must consider how all cell types within the central nervous system are influenced by the neuroendocrine system. This review article discusses the effects of stress and sex steroids on the macroglia: astrocytes and oligodendrocytes. Glia are involved in shaping the synapse through the regulation of neurotransmitter levels and energy resources, making them essential contributors to neural dynamics following stress. As the role of glia in neuromodulation has become more apparent, studies exploring the mechanisms by which glia are altered by stress and steroids will provide insight into sex differences in animal models. These insights will facilitate the optimization of animal models of psychiatric disorders and development of future therapeutic targets.

## Introduction

Despite their variety and vast numbers within the central nervous system (CNS), glial cells were initially considered to function only in a supportive capacity to the electrically excitable neurons. However, following decades of research, glial cells have emerged as active contributors to, and modulators of, neuronal activity with functions in neurodevelopment and neuromodulation through the release of gliotransmitters (substances released by glia to facilitate communication) that continuously shape neuronal activity (Allen and Barres, 2005). There are two main classifications of glial cells within the CNS, macroglia and microglia. Two of the major macroglia include astrocytes and oligodendrocytes (Zhou et al., 2020). Astrocytes make up the majority of glial cells within the brain (Eroglu and Barres, 2010). A single astrocyte process may contact up to 100,000 synapses, allowing for direct contact and the ability to influence synaptic communication. Along with microglia, astrocytes are considered to be essential immune cells within the brain, and are involved in neuroinflammation and neurodevelopment through the promotion and maintenance of synapses (Schwarz and Bilbo, 2012). Oligodendrocytes are responsible for producing myelin within the CNS, providing insulation to axons, and increasing the speed of neuronal action potential propagation (Edgar and Sibille, 2012). Increased understanding of the intimate relationship of glia with the synapse has raised the question of how glia may regulate neuronal abnormalities and the development of disorders, as well as serve as potential therapeutic targets (Halassa et al., 2007).

Given the central role of glial in CNS function, it is essential to develop a complete understanding of their contribution to normal and pathological outcomes. One domain in which glial dysfunctions have been implicated is the development of neuropsychiatric disorders, such as anxiety and depression. This may not be surprising considering that canonical pathways dysregulated in these mood disorders include glutamate homeostasis and inflammation, both of which are tightly regulated by glial cells (Kalivas, 2009; Bilbo et al., 2012). Additionally, glia pathology has been recorded in patients that suffered from major depressive disorder (MDD; Miguel-Hidalgo et al., 2000; Rajkowska and Stockmeier, 2013), and astrocyte-oligodendrocyte communication deficits have been implicated in depression, as shown by decreased glia coupling in postmortem brain tissue of male-depressed suicide victims (Tanti et al., 2019). Although complex neuropsychiatric disorders like anxiety and depression cannot be recapitulated in other animals (Dalla et al., 2010), advances in understanding the neurobiology of depression and anxiety have been facilitated by examination of models of chronic stress, a known catalyst of depression and anxiety in humans (Carr et al., 2013). Therefore, this review will focus largely on the impact of chronic stressors on glia biology and function. Finally, we view glia through the lens of sex differences and examine gaps in understanding. While the number of studies evaluating the role of glia in neuropsychiatric disorders has increased, a full understanding of sex differences within these cell-types requires further examination. Women are twice as likely to suffer from anxiety and depression as men (Albert, 2015). However, neuroscience has extensively utilized male rodents in preclinical studies (Mamlouk et al., 2020), including animal models of anxiety- and depressive-like behaviors. The under-representation of females in both clinical and preclinical research leaves a significant gap in the understanding of the biology of mood disorders. Because the topic of sex differences and microglia has been extensively reviewed elsewhere (Bilbo et al., 2012; Bekhbat and Neigh, 2018; Rainville and Hodes, 2019), this review focuses on the macroglia: astrocytes and oligodendrocytes.

## Primer on Steroids and The Nervous System

We begin with a brief overview of the influence of steroids in the CNS. Steroids are a class of hormones which are highly lipophilic and are synthesized on demand both by endocrine organs and in the central nervous system (Schmidt et al., 2008; Diotel et al., 2018). The most studied class of steroid receptors are the nuclear receptors which act as transcription factors and thereby can exert profound and long-lasting effects within the brain and periphery (Lösel and Wehling, 2003). Steroids are further subdivided into corticosteroids and sex steroids. The corticosteroids include mineralocorticoids and glucocorticoids and are critical in modulation of circadian functions, such as sleep and feeding behavior (Oster et al., 2006; Dickmeis, 2009), and the response to physical and psychological stressors (Jauregui-Huerta et al., 2010). The sex steroids include progesterone, estrogen, and testosterone. All types of sex steroids exist in both sexes. In addition to the influence of sex steroids over reproductive behaviors and secondary sex characteristics (Morris et al., 2004), sex steroids also influence a wide range of normal and pathological functions unrelated to reproduction.

Chronic and traumatic stress influence the manifestation and progression of mood disorders (McEwen, 2007); therefore, we begin by discussing the interactions among glia and glucocorticoids. Disruptions in homeostasis, whether physical or perceived, activate the hypothalamic-pituitary-adrenal (HPA) axis. This leads to an increase in circulating glucocorticoids (GCs) in the bloodstream that bind to glucocorticoid (GR) and mineralocorticoid receptors (MRs) throughout the organism (McEwen, 2007). HPA axis activation is a normal and healthy response to a perceived threat or physical perturbation; however, continuous HPA axis activation during chronic stress can evoke maladaptive consequences within stress-sensitive brain regions as well as in other organ systems. Although GR binds with lower affinity than MR, GR appears to be more complicit in chronic stress-induced effects. The pervasive impact of GR is at least in part driven by the genomic impact of GR such that activation of the GR can lead to transcriptional changes of up to 10% of genes through glucocorticoid response elements (Jauregui-Huerta et al., 2010). The robust influence of GR is regulated by multiple chaperones and co-chaperones and the effects can be cell-type specific. GR expression has been demonstrated on glia cells within the brain (Vielkind et al., 1990; Bohn et al., 1991). Engagement of the transcriptional influences of steroids is also evident in macroglia. In primary oligodendrocytes cultures isolated from male and female mice, steroid hormones differentially influenced cell survival (Swamydas et al., 2009). Cultured astrocytes respond to corticosterone application with an altered expression of 141 mRNAs, an effect that was attenuated by co-administration with the GR antagonist RU486 (Carter et al., 2012). Additionally, corticosterone treatment in rats resulted in decreased expression of the astrocyte enriched structural protein glial fibrillary acidic protein (GFAP; O’Callaghan et al., 1989; Nichols et al., 1990).

Sex steroids are present in both sexes and differ in concentrations and timing of developmental surges (McCarthy et al., 2002). In addition to the well-known functions of sex steroids in reproductive behaviors and secondary sex characteristics, these steroids catalyze a range of changes in physiology through neuronal actions (McEwen and Parsons, 1982) and influence glial development (McCarthy et al., 2002; Marin-Husstege et al., 2004) and function (Kuo et al., 2010). Importantly, sex steroids and GCs also interact with one another and influence the impact of one another to influence both physiology and behavior (Conrad et al., 2004; Hiroi and Neumaier, 2006) illustrating the centrality of considered sex differences in the examination of stress and related neuropsychiatric disorders. This is particularly important in the context of glial biology because, in addition to the impact of sex steroids on glia through estrogen receptors expressed on both astrocytes and oligodendrocytes (Azcoitia et al., 1999; Takao et al., 2004), glia also influence sex steroid function in that they provide a central source of steroid synthesis. Although peripheral sources of steroids (i.e., ovaries, testes, and adrenals) exert substantial influence in the CNS due to their highly lipophilic nature which facilitates the crossing of the blood-brain barrier, neuroactive steroids can be generated centrally from cholesterol synthesized by astrocytes and oligodendrocytes (Arbo et al., 2016). Thus, sex steroids are positioned to influence a plethora of functions in the brain that are outside the realm of reproductive behaviors.

## Astrocytes

The role of astrocytes in neural function, pathology, and repair has provided foundational information for studying the possible role of glia in neuropsychiatric disorders. For instance, progesterone and estrogen have demonstrated neuroprotective effects following injury, inflammation, and stress at the level of glia (Garcia-Ovejero et al., 2005; Arbo et al., 2016). Even in the absence of injury, sex steroids influence astrocyte function. The estrous cycle stage of female rats is associated with glia process ensheathment of synaptic terminals within the arcuate nucleus, with ensheathment decreasing during estrus compared to proestrus (Olmos et al., 1989). Further, the relationship between astrocytes and sex steroids is bidirectional. Glial cells express estrogen receptors and can produce estrogen, thus they can be the driver of estrogen’s protective effects within models of CNS disease (Arevalo et al., 2010). For example, ERα located on astrocytes are responsible for the protective effects of estrogen within a model of autoimmune encephalomyelitis, not neuronal ERα, as seen by decreased levels of macrophage and T-cells of ERα ligand treated animals (Spence et al., 2011).

Although not as easily defined as neural injury studies, a relationship between astrocytes, steroids, and neuropsychiatric disorders has begun to be established. Altered expression of astrocyte density, as seen in post-mortem human tissue, has been implicated in the development of anxiety and depression (Rajkowska and Miguel-Hidalgo, 2007). A similar relationship has been detailed using animal models. Chemically induced deficits of the astrocyte population within the prefrontal cortex (PFC) using the astrocyte toxin L-alpha-aminoadipic acid (L-AAA) induce depressive-like behaviors in rodents (Banasr and Duman, 2008). In addition, exogenous administration of corticosterone, the primary glucocorticoid in rodents, leads to a decrease in the astrocyte structural protein GFAP mRNA (Nichols et al., 1990) and protein (O’Callaghan et al., 1989) within the hippocampus, a stress-sensitive brain region. Changes in GFAP-immunoreactivity (GFAP-IR) may indicate less coverage of the synapse, suggesting a relationship between glucocorticoids and synaptic activity. Changes in cell proliferation rate within the adult CNS can also contribute to altered expression of GFAP positive cells, as seen in male mice that underwent chronic stress and expressed a decreased number of newborn astrocytes in the hippocampus compared to controls (Dioli et al., 2017). In addition, comparison of GFAP-IR in the male Wistar-Kyoto (WKY) rat, which is commonly used as a model in studies of anxiety-like and depressive-like behaviors due to heightened stress-sensitivity, to male Sprague-Dawley rats, found that overall GFAP expression was lower in the PFC, basolateral amygdala (BLA), and hippocampus of the WKY rats (Gosselin et al., 2009). This lower expression of GFAP in stress-sensitive brain regions of the WKY strain suggests that the magnified stress-induced behaviors observed may be due to basal differences in the astrocyte profile within these regions.

While the preceding studies did not include the variable of sex, differences in astrocyte complexity may vary by sex. Ovariectomized (OVX) female rats given estradiol display an increase in GFAP surface density compared to OVX controls in the hippocampus and globus pallidus (Trangue et al., 1987) demonstrating the ability of estradiol to influence astrocytic proteins. In addition, GFAP-IR in the hippocampus of adult Wistar rats was greater in the CA1 region in females, with males displaying greater reactivity in CA3 (Conejo et al., 2003). These findings demonstrate baseline differences in a marker of astrocyte complexity which may be reflective of sex differences in astrocyte phenotype in stress-sensitive brain regions. Further, animal models of stress show sex differences in astrocyte complexity in the medial PFC (mPFC) following chronic stress, results that were sex steroid dependent (Bollinger et al., 2019). This suggests that sex steroids have direct actions on glia activity and may drive sex differences in the glial response to stress, which could facilitate sex differences in behavioral outcomes following stress exposure.

Astrocytes have an established role in neurotransmitter modulation. Importantly, astrocyte-mediated regulation of glutamate basal tone and dysregulation of glutamate homeostasis may contribute to the potentiation of anxiety and depression (Blacker et al., 2019). To this end, the astrocyte enriched glutamate transporter GLT-1 has decreased expression in postmortem tissue obtained from individuals diagnosed with MDD (Choudary et al., 2005; Rajkowska and Stockmeier, 2013). Similarly, preclinical research suggests a role of GLT-1 in behaviors related to mood disorders. Knockout of the GLT-1 gene in astrocyte using male transgenic GFAP-Cre mice results inincreased anxiety-like behaviors in the elevated plus maze (Jia et al., 2020). However, the effects of GLT-1 are region dependent such that deletion of GLT-1 within the lateral habenula increased depressive-like behavior as measured by social interaction and behavior in the novelty-suppressed feeding paradigm (Cui et al., 2014). The brain region specific effects of GLT-1 may also be sex specific. Male and female Long-Evans rats exposed to chronic stress displayed reductions in GLT-1 expression (Rappeneau et al., 2016). However, females had decreased GLT-1 and GFAP mRNA within the PFC and nucleus accumbens (NAc), whereas, males displayed reductions in GLT-1 only in the striatum. Furthermore, comprehensive evaluation of overall glutamate homeostasis in male mice that underwent varying lengths of stress suggests that neuronal and glia protein expression are altered (Nasca et al., 2017). Unfortunately, female mice were not included in this study leaving a gap in knowledge regarding sex differences in glutamate homeostasis following stress which will be important to address given that evidence for differential glutamate homeostasis as a function of sex is evident from postmortem human studies. Analysis of ionotropic and metabotropic glutamate receptor expression in postmortem tissue reveals that females with MDD have higher expression of both receptor classes in the dorsolateral PFC, while males with MDD only display variation in the glutamate metabotropic receptor 5 (GRM5) expression (Gray et al., 2015). While this study focused on neuronal proteins, it suggests there may be sex differences in glutamate homeostasis during the clinical state of depression. Given that astroglia are essential in maintaining glutamate homeostasis and astrocyte-enriched proteins that regulate glutamate clearance are differentially altered in males and females following stress, a comprehensive evaluation of the role of astrocyte enriched protein expression in females following stress will be foundational in building an understanding of the role of astrocytes in the regulation of glutamate homeostasis in the context of both normal brain function and neuropsychiatric disorders.

## Oligodendrocytes

White matter consists of oligodendrocytes that ensheath axons and allow neurons to propagate action potentials (Bonnefil et al., 2019). Similar to astrocytes, our understanding of oligodendrocytes and their relationship to steroids was bolstered by the study of neural injury. Examination of a model of autoimmune encephalomyelitis demonstrated that estradiol promotes oligodendrocyte myelin protein survival (Offner and Polanczyk, 2006). Again in line with astrocytes, evidence exists to indicate a relationship between oligodendrocytes and neuropsychiatric diseases. Reductions in white matter are visible in patients with MDD using neuroimaging (Coloigner et al., 2019). Analysis of the anterior cingulate cortex from male victims of suicide with a history of active depression found decreased gap junction coupling between oligodendrocytes and astrocytes, compared to controls (Tanti et al., 2019). Although causality cannot be assessed in this type of study, animal models can be leveraged to investigate how social experience may alter oligodendrocytes and myelinationcreating a risk factor for developing a neuropsychiatric disorder (Toritsuka et al., 2015). Chronic stress in male mice caused morphological changes of oligodendrocytes in the corpus callosum, including increased length of the paranode and juxtaparanode regions and upregulation of multiple adhesion molecules (Miyata et al., 2016) suggesting compromised function. Following chronic social defeat of male mice, the percent area in the NAc covered with myelin basic protein (MBP) is decreased along with the altered length of myelin segmentation in the mPFC (Bonnefil et al., 2019). In the same study, a demyelination compound was directly targeted at the mPFC and decreased social preference in male mice demonstrating that decreasing myelination alone can lead to altered social behavior, similarly to the depletion of astrocytes in the PFC leading to depressive-like behaviors (Banasr and Duman, 2008). Further, male mice that underwent chronic variable stress displayed increased depressive-like behaviors and oligodendrocyte transcriptional changes in the PFC, NAc, amygdala, and corpus callosum (Liu et al., 2018). Thus, changes in oligodendrocyte function are not limited to social stress alone—at least for male organisms as all of the studies discussed excluded females from the analysis.

Despite the frequent exclusion of female subjects from research studies, there is substantial evidence for sex differences in oligodendrocytes. The density and proliferation of oligodendrocytes vary between male and female mice, with adult castrated male mice displaying similar proliferation in the corpus callosum as females which supports a causative role of sex steroids in oligodendrocyte proliferation (Cerghet et al., 2006). This influence of sex on oligodendrocyte proliferation has been confirmed *in vitro* as demonstrated by greater expression of genes associated with oligodendrocyte proliferation in primary cultures obtained from neonatal female rats compared to males (Yasuda et al., 2020). In addition to basal differences, sex differences in oligodendrocyte markers have been reported following acute stress with differentially altered MBP levels in male and female mice. Male mice displayedincreased levels of MBP in the amygdala and hippocampus 12 days after the stressor (Breton et al., 2020). Conversely, females did not have altered MBP levels until 2 months after the stressor and exhibited increases in the PFC, amygdala, and hippocampus.Thus, the temporal influence of stress on oligodendrocytes differs between the sexes and couldrepresent an important and understudied mechanism driving sex differences in neuropsychiatric diseases.

## Antidepressants and Macroglia

There is robust evidence to support the role of glial cells in the development of neuropsychiatric disorders as identified in human post-mortem pathology and the rodent studies discussed. This relationship is further supported by evidence that glial cells are responsive to the influence of some antidepressant treatments. For instance, astrocyte enriched gap junction protein connexin 43 is lowered by chronic unpredictable stress, but the administration of fluoxetine or duloxetine recovered expression deficit in male rats (Sun et al., 2012). In addition, the antidepressant clemastine rescues behavioral deficits and restores MBP coverage in the PFC of socially isolated male mice (Liu et al., 2016). However, antidepressants are not universally efficacious in reversing stress effects on macroglia as evidence of the failure of citalopram to prevent stress-induced reductions of GFAP in astrocytes of male rats (Araya-Callís et al., 2012). These studies did not consider females and illustrate that only partial responsivity of glia to current antidepressants exists. This represents both an opportunity to directly target macroglia in the treatment of mood disorders and the tremendous need to consider females in these assessments.

## Conclusions

**Figure 1:**
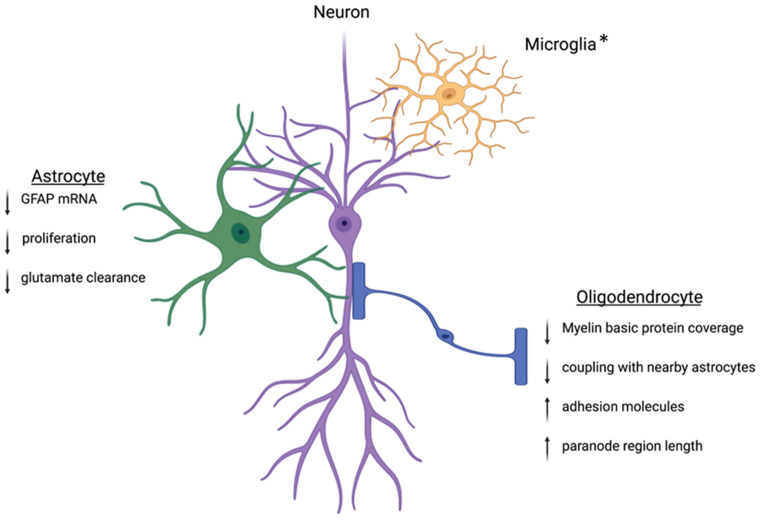
Within the CNS, glia cells are positioned in close contact with neighboring neurons. This positioning allows the glial cells to tightly control the environment and facilitate neuronal communication. Following stress, this regulation can become dysregulated. Key proteins involved in astrocyte function are downregulated leading to decreased cradling at the synapse and altered glutamate homeostasis. Oligodendrocytes will experience downregulation of the key myelin basic protein and altered wrapping at the neuronal axon. Preclinical works suggest protein dysregulation following stress is different between males and females, but more work is needed to understand these mechanisms. *Microglia are intimately associated with the synapse and other nearby glia cells. The interactions between microglia and neurons can be disrupted following stress. While microglia are not discussed in this review, other reviews have extensively outlined the ways microglia contribute to sex differences in the stress response (Bilbo et al., 2012; Bekhbat and Neigh, 2018; Rainville and Hodes, 2019). Created with Biorender.com.

The goal of this mini-review was to highlight the importance of sex in the consideration of the role of macroglia in mental health disorders and altered affective-like behaviors. Glia are involved in shaping the synapse through the regulation of neurotransmitter levels and energy resources, making them essential contributors to neural dynamics following stress. We have highlighted astrocytes and oligodendrocytes, both of which are implicated in the progression of MDD (Figure 1). To date, studies have focused on the role of glia or neuronal proteins in the progression of anxiety and depression; however, opportunities to identify expression variations in multiple cell types within a single condition or preclinical model could elucidate the overall change in tone at the synapse and provide an important contribution to advancing our understanding. Additionally, assessment of glia-glia communication following stress will be a critical area of future study in order to determine how macroglia change the dynamics of neural resources and overall communication. Furthermore, as the role of glia in neuromodulation continues to be elucidated, studies exploring the mechanisms by which glia are altered by stress and steroids will provide insight into sex differences in animal models and inform our understanding of sex differences in the clinical setting. Finally, clarification of macroglia function in health and disease in both sexes will provide the foundation for novel mechanistic interventions that may have the capacity to treat beyond the symptoms and target the biological nexus from which symptoms manifest.

## Author Contributions

GN and AW decided on the topic area together. AW drafted the first draft of the review with guidance from GN. GN revised and edited the review. All authors contributed to the article and approved the submitted version.

## Conflict of Interest

The authors declare that the research was conducted in the absence of any commercial or financial relationships that could be construed as a potential conflict of interest.

## Publisher’s Note

All claims expressed in this article are solely those of the authors and do not necessarily represent those of their affiliated organizations, or those of the publisher, the editors and the reviewers. Any product that may be evaluated in this article, or claim that may be made by its manufacturer, is not guaranteed or endorsed by the publisher.
